# Who gets single tablet regimens (STR), and why?

**DOI:** 10.7448/IAS.17.4.19777

**Published:** 2014-11-02

**Authors:** Laura Tarrier, Stephen Kegg

**Affiliations:** GUM and HIV Medicine, Trafalgar Clinic, Queen Elizabeth Hospital, London, UK

## Abstract

**Introduction:**

The BHIVA guidelines now feature three single tablet regimens (STRs) as recommended treatments for HIV-positive people new to therapy [1]. They are popular with patients and are attractive in a number of clinical scenarios. We sought to determine how our use of STRs had developed over a three-year period, and how the decision to opt for an STR was made****.

**Methods:**

Retrospective case-note review and database interrogation of all patients starting anti-retroviral therapy (ART) from 1st March 2011 to 31st March 2014.

**Results:**

215 patients started ART. 58% (125/215) were black-African and 47% (100/215) were female. Median CD4 at baseline was 272 cells/µL (range 1-1044 cells/µL). 69 (32%) had a viral load (VL) >100,000 copies/mL. 7 individuals had evidence of transmitted drug resistance. 88 patients started an STR. Two tested positive for HLAB5701. None had a 10 year CVS risk score of >20% and 36% had a baseline VL >100,000 copies/mL. 127 patients started a non-STR regimen, 29% had a VL >100 000 copies/mL and none had an elevated CVS risk. Information regarding baseline renal function and HLAB5701 will be provided at the conference. The use of STRs increased over the three years (25% 2011–12; 57.1% 2012–13; 44% 2013–14). There was no difference in STR prescribing between men and women. In men, heterosexual male patients were more likely to be prescribed an STR than MSM males (54% versus 43%, p 0.005). In 43% (38/88) patients had indicated a preference for an STR and 32/88 expressed a preference for a particular drug. 2% (2/88) requested to be on the same treatment as a partner. In those patients who expressed an interest in a particular drug, four had received information from a friend or partner. In 33% (29/88) cases an STR was felt by the clinician to be the best option, largely based on concerns around pill-burden and adherence (31%), viral load (16%) and renal or cardiac risk (7%).

**Conclusions:**

Use of STRs is increasing. This is not driven on in our cohort by cardiovascular risk of HLAB5701 carriage but by patient and clinician preference, and to a lesser extent by higher baseline viral loads.
